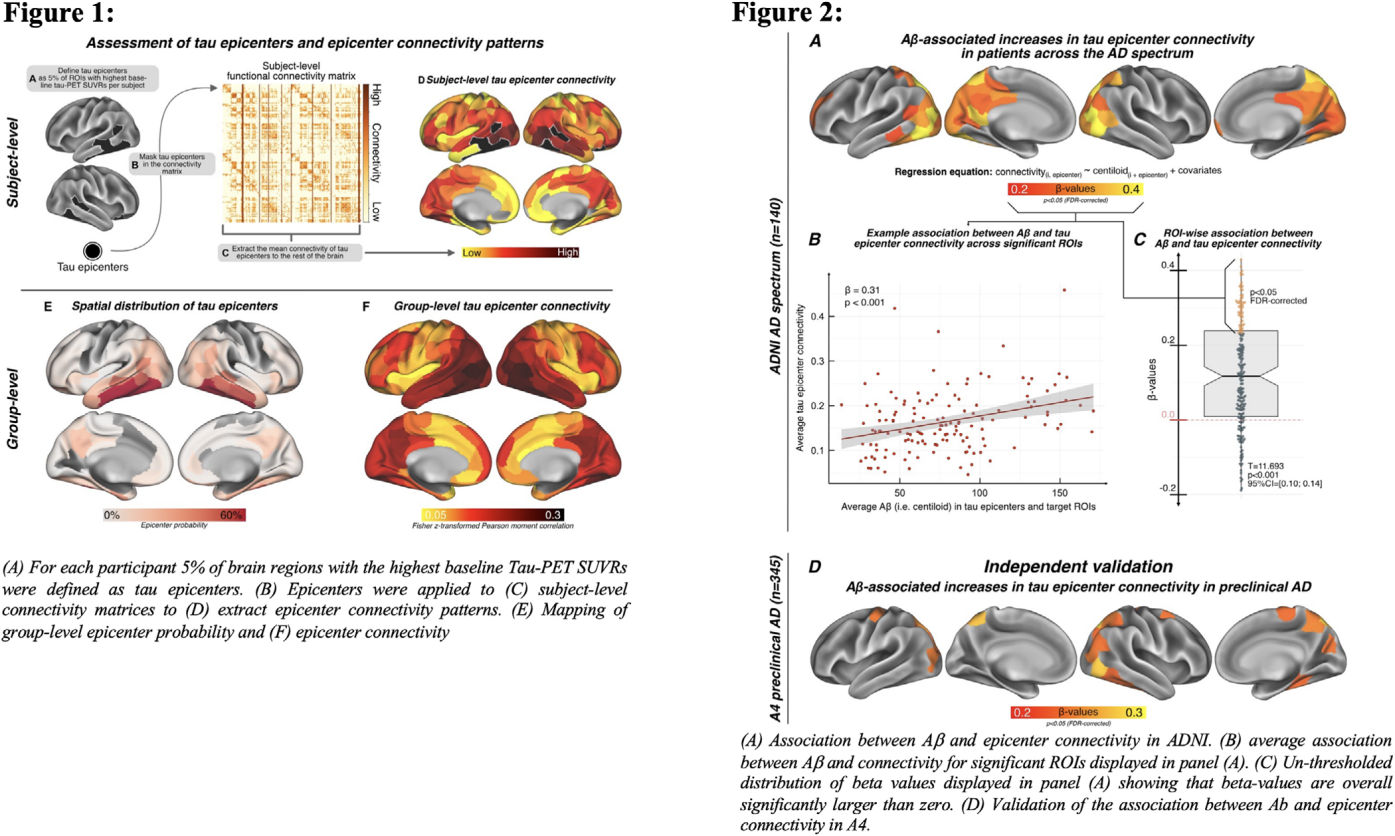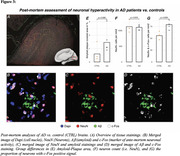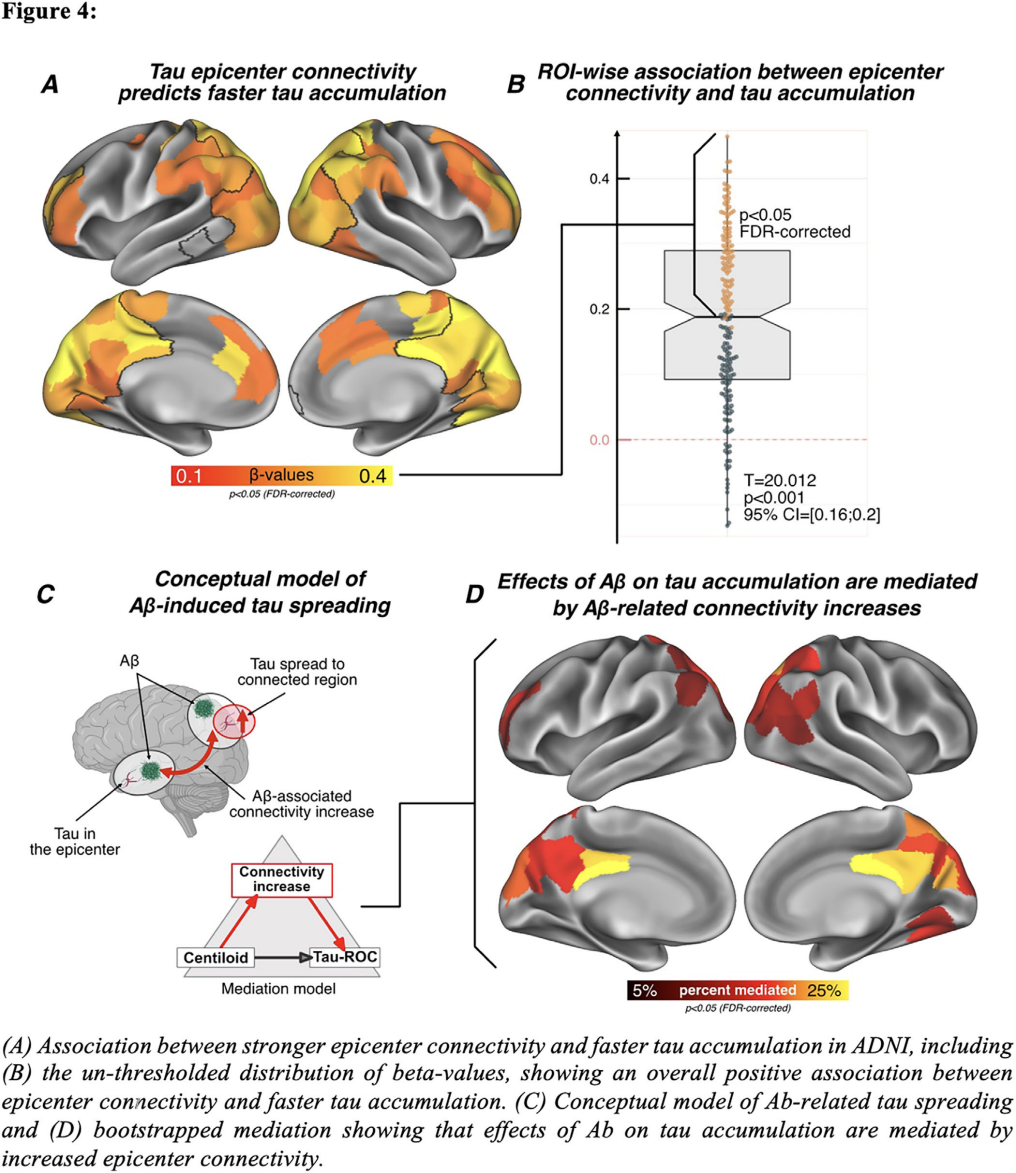# Amyloid‐associated hyperconnectivity drives tau spreading across connected brain regions in Alzheimer’s disease

**DOI:** 10.1002/alz.094374

**Published:** 2025-01-09

**Authors:** Sebastian Niclas Roemer, Fabian Wagner, Lisa Evangelista, Boris‐Stephan Rauchmann, Amir Dehsarvi, Anna Steward, Anna Dewenter, Davina Biel, Zeyu Zhu, Julia Pescoller, Mattes Gross, Robert Perneczky, Maura Malpetti, Michael Ewers, Michael Schöll, Martin Dichgans, Günter Höglinger, Matthias Brendel, Sarah Jäkel, Nicolai Franzmeier

**Affiliations:** ^1^ Department of Neurology, University Hospital, LMU, Munich, Bavaria Germany; ^2^ Institute for Stroke and Dementia Research (ISD), University Hospital, LMU, Munich, Bavaria Germany; ^3^ Institute for Stroke and Dementia Research, LMU University Hostpital, Munich Germany; ^4^ Department of Psychiatry and Psychotherapy, University Hospital, LMU Munich, Munich Germany; ^5^ Institute for Stroke and Dementia Research (ISD), University Hospital, LMU, Munich Germany; ^6^ Institute for Stroke and Dementia Research (ISD), LMU University Hospital, Munich, Munich (Bavaria) Germany; ^7^ Imperial College London, London United Kingdom; ^8^ Department of Clinical Neurosciences and Cambridge University Hospitals NHS Trust, University of Cambridge, Cambridge United Kingdom; ^9^ German Center for Neurodegenerative Diseases (DZNE), Munich, Bavaria Germany; ^10^ Wallenberg Centre for Molecular and Translational Medicine, University of Gothenburg, Gothenburg Sweden; ^11^ German Center for Neurodegenerative Diseases (DZNE), Munich Germany; ^12^ Department of Neurology, Klinikum der Ludwig‐Maximilians Universität München, Munich Germany; ^13^ Department of Nuclear Medicine, University Hospital, LMU Klinikum, Munich, Bavaria Germany; ^14^ Institute for Stroke and Dementia Research (ISD), LMU University Hospital, Munich Germany; ^15^ Institute for Stroke and Dementia Research, Ludwig‐Maximilians‐Universität München, LMU München, Munich Germany

## Abstract

**Background:**

In Alzheimer’s disease, Aß triggers tau spreading which drives neurodegeneration and cognitive decline. However, the mechanistic link between Aß and tau remains unclear, which hinders therapeutic efforts to attenuate Aß‐related tau accumulation. Preclinical research could show that tau spreads across connected neurons in an activity‐dependent manner, and Aß was shown to trigger neuronal hyperactivity and hyperconnectivity. Therefore, we hypothesized that Aß induces neuronal hyperactivity and hyperconnectivity, thereby promoting tau spreading from initial epicenters across connected brain regions.

**Methods:**

From ADNI, we included 140 Aß‐positive subjects across the AD spectrum plus 69 Aß‐negative controls, all with baseline amyloid‐PET, 3T resting‐state fMRI and longitudinal Flortaucipir tau‐PET data. For validation, we included cross‐sectional tau‐PET, amyloid‐PET and resting‐state fMRI data of 345 preclinical AD patients from A4. PET and fMRI data were parceled into 200 cortical ROIs, ROI‐wise longitudinal tau‐PET change rates were computed using linear mixed models. Resting‐state fMRI connectivity was computed across the 200 ROIs. Subject‐specific tau epicenters were defined as 5% of ROIs with highest baseline tau‐PET. Further, we included post‐mortem brain tissue from 5 AD patients vs. 4 controls stained for Aß and c‐Fos, i.e. a marker of ante‐mortem neuronal activity.

**Results:**

In the AD spectrum cohort, we confirmed that Aß induces hyperconnectivity of temporal lobe tau epicenters (Fig.1) to posterior brain regions that are highly vulnerable to tau accumulation in AD (Fig.2A‐C). This was fully replicated in the validation cohort of preclinical AD patients with low cortical tau‐PET, suggesting that the emergence of Aß‐related hyperconnectivity precedes neocortical tau spreading (Fig.2D). Supporting that Aß‐associated fMRI‐based hyperconnectivity may mirror neuronal hyperactivity, we found that neurons in AD post‐mortem tissue expressed higher levels of c‐Fos compared to controls, i.e. a Calcium‐sensitive marker of ante‐mortem neuronal activity (Fig.3). Lastly, using longitudinal tau‐PET, we confirmed that Aß‐related connectivity increases of the tau epicenters to posterior brain regions mediated the effect of Aß on tau accumulation and triggered faster tau spreading (Fig.4).

**Conclusions:**

Our translational results suggest that Aß promotes tau spreading via increasing neuronal activity and connectivity. Therefore, Aß‐associated neuronal hyperexcitability may be a promising target for attenuating tau spreading in AD.